# Long-lasting insecticidal net ownership and malaria infection by socio-economic status: a cross-sectional household study in an area along Lake Victoria, Kenya

**DOI:** 10.1186/s12936-025-05528-x

**Published:** 2025-10-22

**Authors:** Hanako Iwashita, Sachiyo Nagi, Felix Bahati, Wataru Kagaya, Peter S. Larson, James Kongere, Bernard N. Kanoi, Reiko Hayasaka, Tomohiko Sugishita, Jesse Gitaka, Akira Kaneko

**Affiliations:** 1 Department of Hygiene and Public Health, Tokyo Women’s Medical University, 8-1 Kawada-Cho, Shinjuku-Ku, Tokyo, 162-8666 Japan; 2 Department of Parasitology, Institute of Tropical Medicine, Nagasaki University, Nagasaki, 852-8523 Japan; 3 Center for Research in Tropical Medicine and Community Development (CRTMCD), Nairobi, Kenya; 4 Osaka International Research Center for Infectious Diseases/ Graduate School of Medicine, Osaka Metropolitan University, 1-4-3, Asahimachi, Abeno-Ku, Osaka, 545-8585 Japan; 5 Department of Eco-Epidemiology, Institute of Tropical Medicine, Nagasaki University, 1-12-4 Sakamoto, Nagasaki, 852-8523 Japan; 6 Injury Prevention Center, University of Michigan, 2800 Plymouth Road, Suite B10-G080, Ann Arbor, MI 48109-2800 USA; 7 Department of Epidemiology, School of Public Health, University of Michigan, 1415 Washington Heights, Ann Arbor, MI 48109 USA; 8 Centre for Malaria Elimination, Directorate of Research and Development, Mount Kenya University, 342-01000 Thika, Kenya; 9 ABeam Consulting Ltd, 2-2-1 Yaesu, Chuo-Ku, Tokyo, 104-0028 Japan; 10 Yakushima Onoaida Clinic, 136-6 Onoaida, Yakushima, Kagoshima 891-4404 Japan; 11 Department of Microbiology, Tumor and Cell Biology, Karolinska Institutet, 17177 Stockholm, Sweden; 12 Centre for Research in Infectious Diseases, Directorate of Research and Development, Mount Kenya University, 342-01000 Thika, Kenya

**Keywords:** Long-lasting insecticidal nets (LLIN), Socio-economic status (SES), Indoor residual spraying (IRS)

## Abstract

**Background:**

This study focused on the importance of long-lasting insecticide treated nets (LLINs) in malaria control in a study area where socio-economic disparities are widening. The objective was to assess the effectiveness of LLIN ownership when nets were available for no more than two people, controlling for differences in socio-economic status (SES). It was hypothesized that LLIN effectiveness would differ by SES and that LLIN effectiveness should be analysed with adjustment for differences in SES.

**Methods:**

A household level survey was conducted in an area in the Lake Victoria region in Suba North Sub-County, Homa Bay County, Western Kenya between June and September 2021. Through the household survey, the number of people living in the home, the number of LLINs, and demographic data were recorded. The ratio of the number of people reporting sleeping in the house to the total number of LLINs in the home was calculated. Through a school-based malaria survey, researchers administered blood-spot, PCR tests for *Plasmodium* infection. Community workers linked individual malaria tests to homes that were involved in the household survey through names and geographic identifiers. A generalized linear model (GLM) tested the associations between household parasitaemia risk in children and the ratio of people to LLINs, stratifying on asset-based household level SES measures.

**Results:**

The association between sufficient LLIN ownership and household malaria infection status was analysed across SES groups. In middle SES households, sufficient LLIN ownership was significantly associated with lower malaria infection status compared with insufficient LLIN ownership (OR 0.32, 95% CI 0.12–0.92). In the low SES group, a similar trend was observed, although it was not statistically significant (OR 0.59, 95% CI 0.15–2.91). When middle and low SES groups were combined, sufficient LLIN ownership remained significantly associated with lower incidence (OR 0.37, 95% CI 0.17–0.87).

**Conclusions:**

The results suggest that promoting the use of one LLIN by no more than two people, as recommended by WHO, can reduce the risk of malaria. Efforts to promote LLINs as an effective means of preventing malaria in children might encourage LLIN compliance and maintain community level control targets. If households have been excluded from LLIN distribution, LLINs should be distributed immediately along with information about their effectiveness. The effectiveness of LLINs varies by region, but strategies to sustain LLIN use should be recognized as contributing to benefits for the entire community.

**Supplementary Information:**

The online version contains supplementary material available at 10.1186/s12936-025-05528-x.

## Background

Long-lasting insecticidal nets (LLINs) have played a critical role in reducing malaria transmission globally. Since the early 2000s, the large-scale distribution and use of LLINs has been associated with substantial declines in malaria morbidity and mortality across endemic regions [1]. LLINs are widely recognized as a fundamental and economical tool for preventing malaria. While World Health Organization (WHO) has promoted integrated vector management (IVM) as a comprehensive approach that includes LLINs, indoor residual spraying (IRS), larviciding, and surveillance, in practice, LLINs and IRS remain the most widely implemented interventions due to their proven impact [2]. Regular and widespread use of LLINs at household level plays an important role in maintaining efforts to reduce malaria.

The Government of Kenya Ministry of Health began mass, cost-free distribution of LLINs in the Lake Victoria region in Western Kenya more than 15 years ago [3]. According to the 2020 Kenyan Malaria Indicator Survey (KMIS), having one LLIN for every two people is considered sufficient; LLINs have been distributed in Kenya using various approaches, including mass net distribution campaigns (by National Malaria Control Programme), antenatal care clinics (ANC) for pregnant women, and child welfare clinics (CWCs) for children under one year of age [4]. In 2020, 49% of households in Kenya owned at least one insecticide-treated net (ITN) which were mostly LLINs [4]. The 2022 World Malaria Report indicated that the modelled percentage of the population in Kenya with access to LLINs and number of ITNs were as follows: 61.2% (1,797,075 nets) in 2019, 46.2% (1,349,895 nets) in 2020, 57.4% (17,912,956 nets) in 2022 [5]. According to WHO reports on coverage with either LLIN or IRS in 2021, no African country exceeded 80%. However, Kenya was among those countries that surpassed the next threshold of 50% and is considered one of the countries with relatively high LLIN coverage in Africa [5]. WHO emphasizes not only increasing the distribution of LLINs but also to encouraging long-term household use [6].

In Kenya, where the gap between the rich and the poor is widening [7], the study examined whether households in this study area were able to properly use LLINs and assessed their effectiveness, taking socio-economic disparities into account. To determine the effectiveness of LLINs in this study, two points were ascertained. First, it was assessed whether two or fewer people shared a single LLIN, which is the appropriate use of LLINs recommended by WHO [2]. Second, we considered differences in the effectiveness of LLINs within distinct socio-economic status (SES) groups. At the local level, the risk for malaria and other infectious diseases is higher in households with low SES than in those with high SES [8]. Higher SES groups are more likely to have access to a variety of malaria prevention and treatment resources than lower SES groups [9]. Low SES groups may have access to only limited resources for malaria control and must rely on basic control tools such as LLINs. We hypothesized that LLINs are effective in controlling malaria and that households with lower SES are more likely to rely on free LLINs, indicating higher LLIN effectiveness. Therefore, anticipating that the effect of LLINs would vary with SES. SES was assumed to be the effect modifier in the analysis of the relationship between LLINs as the exposure and malaria as the outcome. An effect modifier is defined as a variable that modifies the relationship between the exposure and outcome. The aim of this study was to understand the effectiveness of LLINs in the study area, specifically investigating whether SES serves as an effect modifier in the association between LLIN use and malaria in this region. In this study, we defined “sufficient LLIN ownership” as the ownership of one net by two or fewer people, using a physical size-based calculation method [10]. In this regard, identifying households that had not received an adequate distribution of LLINs was an additional objective.

## Methods

### Study setting

This study was conducted along Lake Victoria region in Suba North Sub-County, Homa Bay County, Western Kenya. Rainfall patterns in the region are bimodal, with a long rainy season from March to May and a short rainy season from November to December. However, the overall level of precipitation and frequency of rainfall changes from year to year [11]. An increase in malaria has been observed in the 2–4 months lag after rainfall [12]. The main vectors are *Anopheles gambiae *sensu stricto (*s.s*.), *Anopheles arabiensis* and *Anopheles funestus s.s*.[13]. Principal economic activities include fishing and farming [10]. In selecting study participants, two communities were chosen that neighbour each other with similar lifestyles and habits: Mbita Township D and Nyamalandi. The majority of residents belong to the Luo ethnic group [14]. Although Luo is the main language spoken, many also speak English and/or Kiswahili [14].

### Study design

**Fig.1:**
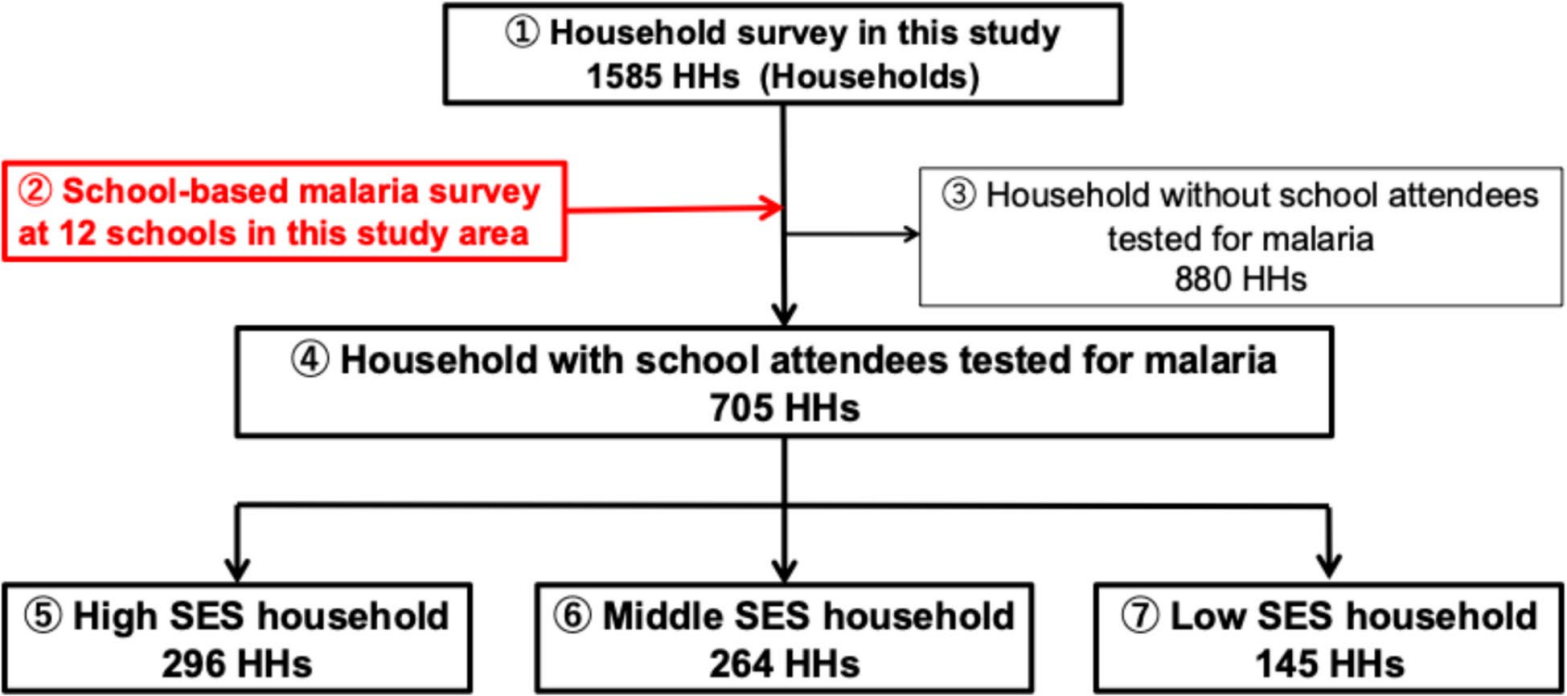
Flow chart of the study sample in Mbita Township D and Nyamarandi

This study was a combination of a cross-sectional household survey and a school-based malaria survey. Households with at least one school-age child with malaria survey results from both surveys were included in the analysis (Fig. 1). Households where malaria testing results were not available for any resident were excluded from this study (Fig. 1, ③).

### Sampling and data collection

Due to ongoing malaria research in this area under the Science and Technology Research Partnership for Sustainable Development (SATREPS) project, this study site was designated as the location for the household survey, and researchers obtained a census of households from local community health workers. All households were included in this study (Fig. 1, ①). Trained research staff visited each household and spoke with an available adult household representative. The goals, risks and benefits of the study were explained and instructions provided on how to contact local staff for more information, if needed. If adult representatives were amenable, research staff asked participants to sign an informed consent form. All information was recorded using REDCap equipped handheld tablet devices [15]. As the local language is Luo, all consent documents and questionnaires were translated from English into Luo and then back-translated to ensure accuracy. In addition, meetings were held with local staff to coordinate the wording of questions in Luo, Kiswahili and English so that there would be no differences in nuances between the languages.

Survey workers conducted the household survey by visiting each household and administering a verbal questionnaire to a single household representative. A representative of the household was defined as the head of the household or a person aged 18 years or older who knew about the household. The survey instrument collected information on the number of LLINs owned by the household, the number of residents in the household who sleep and eat in the same house, whether IRS had been conducted in the past one year, and information on the home. In addition, research staff recorded information on household assets following standards common to survey assessments of SES in developing countries [16]. Survey workers also visually confirmed responses. Survey workers counted the number of LLINs, checked whether the eaves were closed (preventing mosquitoes from entering) or opened (not preventing entering), whether the roof was iron sheeting or not (straw thatching etc.), whether the walls were mud or not (cement, blocks, bricks, stones, iron sheets), and whether the floor was mud/earth or not (cement, blocks, bricks, stones, tiles, wood plank). Specifically, regarding LLIN ownership, the number of LLINs owned and the number of people in the household, as confirmed through the questionnaire and observation, were used. The questionnaire also included the question, "Did you use LLINs yesterday?", but this item was not utilized in the analysis. At the time of the household survey, consent forms were distributed to household representatives of children attending schools and representatives were asked to bring the forms to the school-based malaria survey.

**Fig.2:**
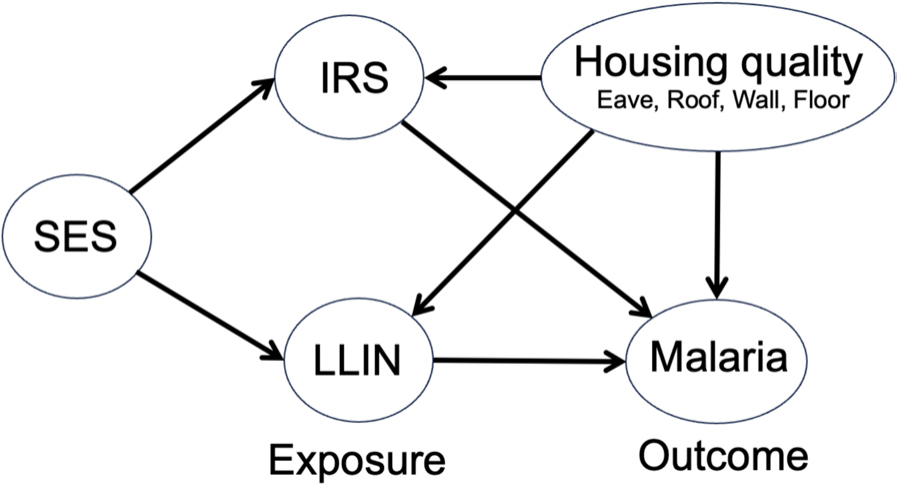
Directed acyclic graph of the relationship between LLIN and Malaria

The school-based malaria survey was conducted in the same regions as the household survey under the SATREPS project, malaria tests were conducted in 12 schools including nursery schools in the study area (Fig. 1, ②). The schedule for the malaria testing was then agreed with the schools and information was shared with all stakeholders, including the children. On the day of the survey, all school-going children whose parents had signed the consent form were invited to participate in the survey. After the malaria PCR test was completed in the laboratory, the results were linked to the household number the child belonged to. All operations were carried out between June and September 2021.

In this study, PCR testing assessed evidence for *Plasmodium* infection in children. For PCR detection, blood samples (70 μL) withdrawn by 75-mm micro-hematocrit capillary tube (Thermo Fisher Scientific, MA, USA) were spotted on Whatman ET31 Chr filter paper (Whatman International, Maidstone, UK). Dried blood spots were stored in individual zipped plastic bags at − 20 ºC. DNA was extracted from a quartered blood spot using the QIAamp Blood Mini Kit (QIAGEN, Germantown, USA) according to the manufacturer’s instructions. *Plasmodium* infection was detected by a nested PCR protocol [17].

## Data collection

### Measurements

#### Exposure: LLIN use

WHO recommends that malaria control programmes aim for a ratio of one LLIN for every two people in a household, including children [2]. In line with this goal, this study created a more nuanced measure based on the physical size of residents. Infants (< 5 years) were treated as 0.3 of a person, a child 5–15 years was 0.5 person, and an adult (above 15 years) is counted as 1 person [10]. The total adjusted number of residents in each household was divided by the number of LLINs they possessed. Following a previous study, this adjusted ratio of people to LLINs was divided into two categories, 2 people or fewer and more than 2 people. In the case of 2 people or fewer, it was defined as "sufficient," and for more than 2 people, it was defined as "insufficient."

#### Outcome: malaria positive household

**Fig.3:**
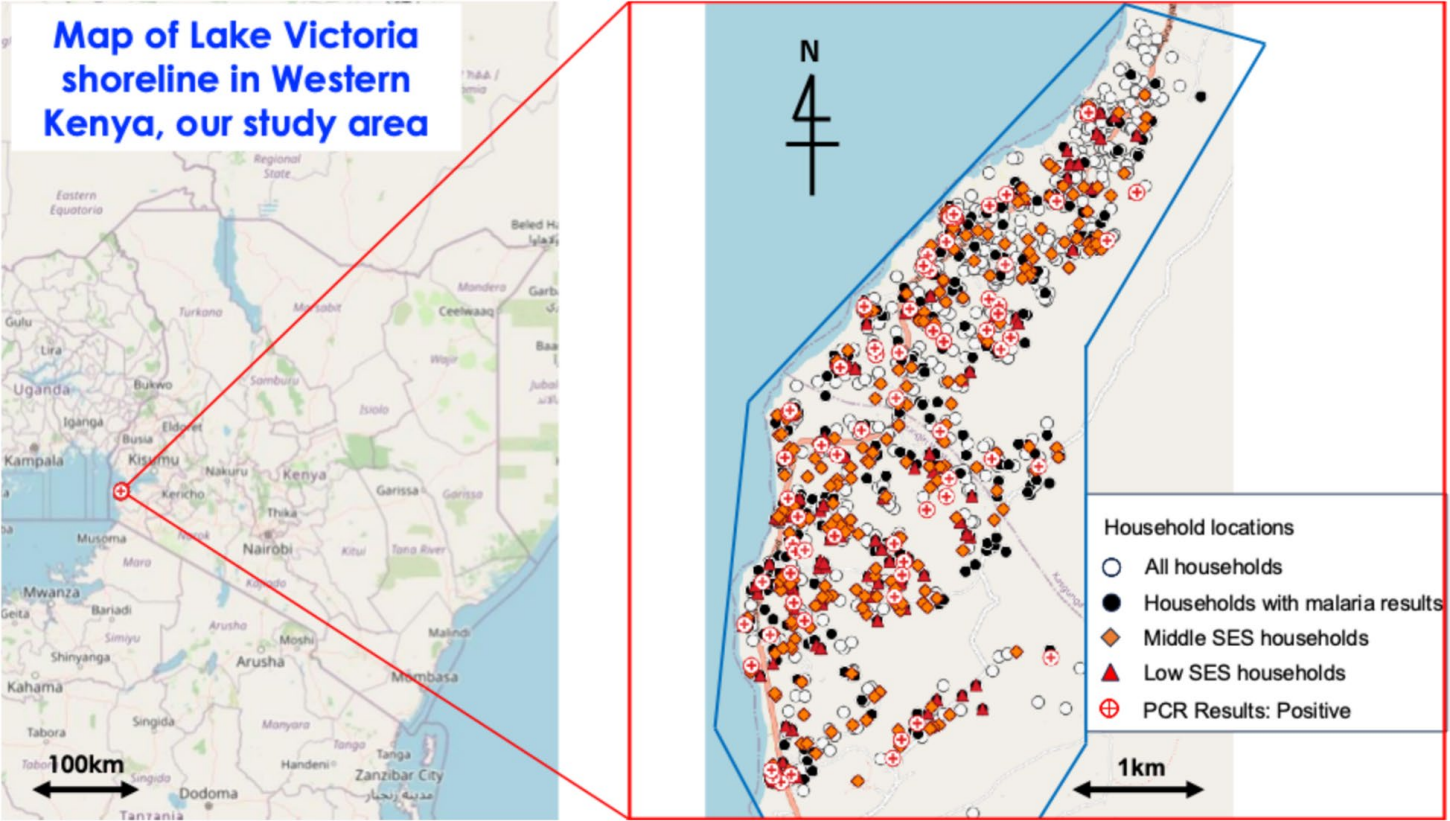
Map of Lake Victoria shoreline in Western Kenya, our study area

The PCR results from the school-based malaria survey were merged with the household data (Fig. 1, ③). Households with one or more malaria-positive children were considered a malaria positive household.

### Effect modifier

The study sought to determine whether SES served as a potential effect modifier on the association between “LLINs ownership” and “malaria positive household”. The present analysis assessed household level SES status using composite, asset-based wealth indices. Household assets were tallied up and weights for each were assigned using multiple correspondence analysis (MCA) methodology [14]. Total continuous SES scores for the entire sample were divided into SES classes using tertiles roughly approximating SES groups. Assets included radio, watch/clock, solar panel, car battery, electricity, television, VCR/DVD player, refrigerator, mobile/telephone, bicycle, motorcycle, car/trucks, boat, land, water tank, roof, wall, floor. SES was divided into 3 levels: low, middle and high.

### Covariates

This study outlined the assumptions regarding the association of variables of interest a directed acyclic graph (DAG) using the Dagitty tool (available at: http://www.dagitty.net/dags.html) (Fig. 2). We included the minimum set of potential confounders as covariates. These included variables such as non-LLIN malaria control measures such as IRS (yes/no) and variables for housing quality: open or closed eaves, type of roof (iron sheer or no), walls (mud or other) and floor type (mud or other).

### Statistical analysis

All analyses were conducted at the household level. Descriptive statistics include the display of percentages for categorical variables and the display of means and standard deviations for continuous variables. Through DAG, the selected variables were analysed using a multivariate generalized linear model (GLM) with binomial distribution, to examine the relationship between malaria-positive household and LLIN ownership. The response variable was a binary variable of malaria-positive household (Yes or No), and the explanatory binary variables included the following; interaction term between LLIN ownership (sufficient or insufficient) and SES (high, middle, low); interaction term between IRS conduct (Yes or No) and SES (high, middle, low), eave (closed or opened); roof (iron sheet or not); wall (mud or other than mud); and, floor (mud/earth or other than mud/earth). In this study, SES was treated as an effect modifier, the interaction term of SES and LLINs and IRS is based on the assumption that these malaria control interventions have different effects at different levels of SES. As a validation step, this study also conducted an analysis in which there was no interaction term and SES, LLINs, and IRS were treated as separate variables and compared to the case with an interaction term. When SES was considered an effect modifier, this study also performed subgroup analyses for each SES level. Odds ratios (OR) and corresponding 95% CI were calculated. When interaction terms showed a meaningful association, GLM were performed for each level of the covariate. R version 4.3.0 (R Core Team, Vienna, Austria) with lme4 package was used for all analyses.

## Results

### Baseline information

In this study area, 1585 households consented to participate (Fig. 1, ①). In the school-based malaria survey conducted in 12 schools, some 1308 school children were tested for malaria by PCR (Fig. 1, ②). Age of participants ranged from 2 to 19 years, with a mean of 10 years and a standard deviation of 3.5 years. Of these school children, 705 households were identified as being included in the survey (Fig. 1, ④). Households without children and households without malaria test results for children were excluded (Fig. 1, ③). These households had fewer residents and fewer LLINs per household compared to the overall household population. In the flowchart in Fig. 1, detailed information for each individual is provided in Supplementary Table 1 and detailed information for each household is provided in Table 1. The location of all houses is shown in Fig. 3. The asset information used to level the SES (Fig. 1, ⑤⑥⑦) is as shown in Supplementary Table 2.

**Table 1:**
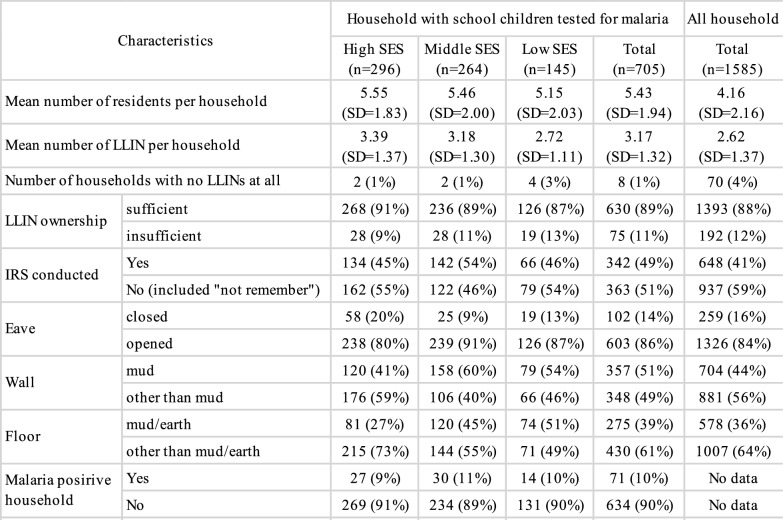
Characteristics of households

Among households with PCR test results (N = 705), 71 households (comprising a total of 80 individuals with a positive test) were categorized as malaria-positive (Supplementary Table 3) and 634 households were categorized as malaria-negative.

### Impacts of LLIN ownership controlling for differences in SES, on malaria infection status

To examine how LLIN ownership was associated with malaria infection status among households from different SES groups, the researchers ran multiple logistic regression models. IRS was included as a possible confounding variable in the models. Interaction terms for LLIN ownership and SES were included in the models. SES was treated as an effect modifier; the interaction terms allowed the researchers to test whether there were different effects of malaria control at different SES levels. The house structure variables (eave, wall and floor) were also included in the analysis. The roof variable was dropped from subsequent analyses because most (99.3%) houses had roofs made of iron sheet: Only 11 had roofs made from other materials (e.g. asbestos shingles, cement, blocks or grass). For the analysis with the interaction term (Table 2), the residual deviance was 445.48 with 693 degrees of freedom and AIC of 469.48. For the analysis with the same variables but without the interaction term (Supplementary Table 4), the residual deviance was 450.89, the degrees of freedom were 697, and the AIC was 466.89. This result suggested that there was no difference between the two and that subgroup analyses by SES level was worthwhile in explaining relationship between outcome, malaria and exposure, LLIN (Table 2).

**Table 2:**
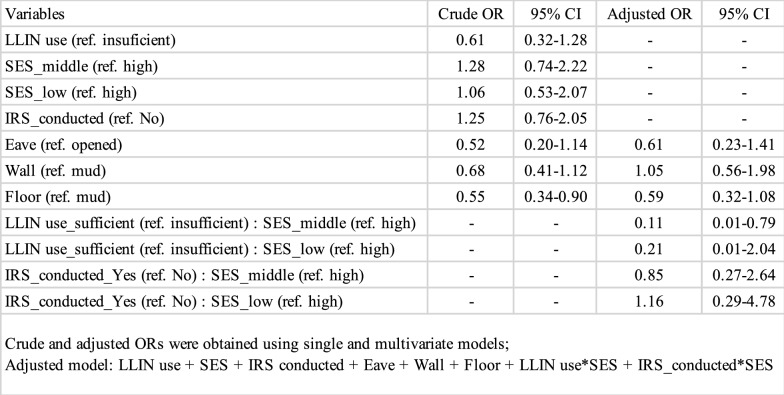
Parameter estimates for the GLM explaining malaria infection status (N = 705)

Crude and adjusted ORs were obtained using single and multivariate models;

Adjusted model: LLIN use + SES + IRS conducted + Eave + Wall + Floor + LLIN use*SES + IRS_conducted*SES

### The impact of LLIN ownership on household malaria at each level of SES

This study analysed the association with malaria infection status at each of the three SES groups (high, middle, low) with the same explanatory variables as in the above analysis. Unlike the above analysis, no interaction terms were used because each model represented only a single SES group. The eaves variable was dropped for the model of the low SES group because there were no malaria-positive households which had closed eaves (See Supplementary Table 5). For the middle SES group, LLIN ownership had a statistically significant association with lower malaria infection status (OR 0.32, 95% CI 0.12–0.92) (Table 3). On the other hand, in the high SES group, there was no evidence to suggest that LLIN ownership was associated with malaria infection status. The actual figures showed only one malaria-positive household in households with insufficient LLIN use, suggesting that it is difficult to determine the effectiveness of LLIN ownership in the high SES group (Grey-coloured cells in Supplementary Table 5). In both the low and middle SES groups, LLIN ownership was observed to be associated with lower malaria infection status. However, while the association was significant in the middle SES group (OR 0.32, 95% CI 0.12–0.92), it was not significant in the low SES group (OR 0.59, 95% CI 0.15–2.91) (Table 3). When excluding the high SES group and combining the medium and low SES groups, malaria positivity was significantly lower among children from households with sufficient LLINs (OR 0.37, 95% CI 0.17–0.87) (Table 3).

**Table 3:**
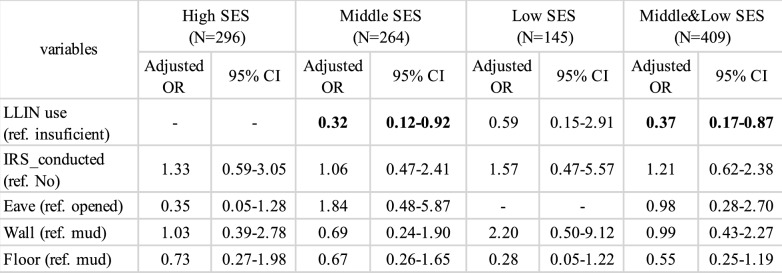
Parameter estimates for the GLM explaining malaria infection status for each SES

“–” indicates that the variable was excluded from the analysis due to its distribution suggesting insufficient analytical value. Bold values indicate statistically significant results.

## Discussion

This study aimed to evaluate the effectiveness of LLINs at local level. LLIN ownership tended to be associated with malaria infection status of household. Households with lower SES may be at higher risk of malaria and are expected to benefit more from LLINs than households with higher SES due to the lack of other malaria prevention measures. The difference in compliance with LLIN use may vary at the local level. It was considered that higher SES is associated with a greater number of alternative malaria control strategies, leading to a lower reliance on LLINs. Therefore, the hypothesis was proposed that the effect of LLINs would differ by SES, and that SES would be an effect modifier. Although the high SES group had slightly lower malaria infection status compared to other groups (Table 1), these differences were not statistically significant (Table 2). As hypothesized, subgroup analysis revealed more distinct patterns. Specifically, evidence was found of an association between sufficient ownership of LLINs and malaria infection status in the middle SES group, suggesting that LLINs may be effective in this group. In the lowest SES group, however, the association between LLIN ownership and malaria infection status was not statistically significant, likely due to the smaller number of children with available malaria test results in this group compared to the middle SES group. When the middle and low SES groups were combined, LLIN ownership was statistically associated with malaria infection status. For high SES households, the effect of LLINs appears to be negligible. This suggests that the absence of malaria-positive children in the absence of LLIN use may be due to malaria control measures other than LLIN use. During the field survey, anecdotal cases were encountered where households possessed a sufficient number of LLINs but stopped using them after renovating their home to better living conditions, and subsequently their children contracted malaria. These observations, while not systematically collected, highlight real-world behaviours that may not be captured in quantitative data. In such cases, the collected data would indicate that the household has sufficient LLINs, but in reality it suggests low compliance with LLIN usage. Similar patterns of LLIN under-utilization despite ownership have been reported in other studies as well [18].

This study analysed the effectiveness of implementing LLINs as a measure within this framework. In particular, it is essential that the population is accurately informed and educated on the proper use of LLINs, as indicated by WHO. Tamari et al. reported that WHO recommendation of ‘one net for every two people’ is adequate, and that the preventive effects are lost if more than two people use the net [19]. When the questionnaire asked simply in this survey, “Did you use LLINs yesterday?” 94% (665/705) of respondents said they did. However, when an analysis was conducted to examine whether the ownership of the LLINs allowed for use by two or fewer people, the number dropped slightly, and 80.9% (570/705) were considered to be using LLINs appropriately. In this study, body size is taken into account by calculating infants as 0.3 and other children as 0.5, considering how they actually sleep within the household [10]. When calculated in this manner, sufficient net ownership was estimated at 89% (630/705). Public health professionals should ensure adequate distribution of LLINs so that households are not forced to share nets among too many individuals, which can reduce their effectiveness [19]. It is equally important to share tips on how to care for LLINs to maximize the effectiveness of LLINs [20]. If not enough LLINs are distributed, residents cannot use them even if they wanted to. Although the number is small, it is listed in Table 1 as “Number of households with no LLINs at all”. In those cases, LLINs should be distributed as soon as possible. In this study findings indicated that the average number of LLINs per household was lower in low SES than in high SES, although the goal of one LLIN per two people has been achieved. If there is a shortage of LLINs, the LLINs that are available must be properly targeted where they are most needed. Efforts should be made to share reliable information to encourage people to continue using LLINs so that residents can receive the maximum benefit of not only personal protection but also the community effect of maximal coverage [21].

## Limitations

This study had several limitations. First, although malaria transmission is the result of many factors other than those addressed in this study, such as geographical factors and the behaviour of local vector populations, this study only addressed information on vector control measures (IRS, LLINs) in place in the study area. Second, it was not possible to validate responses to questions on LLIN use through observation or the details of how residents care for their LLINs. As a proxy measure for total potential proper use of LLINs, this study could only use the number of family members and the number of LLINs. A third limitation, the malaria infection status of the household was only measured through a PCR test of children; there were no data available on the parasitaemia status of older children and adults. A household was classified as positive if at least one child tested positive for malaria, even if other children tested negative, when the household had malaria test results for multiple children. Since this study was analysed at the household level, some individual cases may have been overlooked. The decision to conduct at the household level, rather than the individual level, was made to improve the stability and reliability of the analysis. This approach enabled the use of more robust household-level data, collected through direct visits and reported by adult members, as opposed to relying on potentially variables data based on children. Nevertheless, there were children who were unable to attend the school-based malaria survey. Finally, although SES is an effect modifier and each SES has a different effect on malaria control, it is possible that there was some factor upstream or downstream of SES in the DAG that made a difference in malaria control. An example of such a factor could be hospital visits when people become ill, and if such a factor could have been measured, a causal relationship could have been inferred based on a more detailed hypothesis.

## Implications

LLINs are effective in preventing malaria transmission, but their protection is limited to areas where they are used, particularly indoors. In fact, even those who use LLINs properly at home can be bitten by malaria-carrying mosquitoes and become infected with malaria if they happen to go outdoors or to other places where LLINs are not used [22]. If people are unaware of the possibility of malaria transmission outside and overly rely on LLINs, their confidence in LLINs is likely to decrease, and many people may become less proactive in complying with LLIN-based interventions. To prevent such a situation, it is important to properly inform people about measures they can take both inside the house, such as using LLINs correctly, and outside the house, such as being aware of malaria-transmitting mosquitoes when going outside at night. This study assessed the effectiveness of LLIN use at local level in this study area. Psychological research shows that people are less likely to engage in sustainable behaviour if they are not aware of the potential positive outcomes of their actions [23]. To mitigate this, it is hoped that the results of this study will be used to raise community awareness about the current effectiveness of LLINs as a malaria control measure, serving as a first step toward maintaining malaria prevention efforts.

## Conclusions

The risk of malaria was found to be lower in households where the ratio of people to nets was appropriate to prevent infection, especially among households with middle and low SES in this study area. Limited to this specific region, these findings should be emphasized not only to local residents, but also to policy makers and many other stakeholders. To maximize the preventive impact of LLINs, policy efforts should prioritize the adequate and equitable provision of nets across all SES groups, with a particular focus on households of lower SES. Although opportunities for free LLIN distribution have already been provided within the community, it is important to continue conveying the message that it is not only essential to possess a LLIN but also to use it consistently. If LLINs have not been distributed to vulnerable populations at risk of malaria transmission, their prompt distribution should be prioritized. Furthermore, information regarding the effectiveness of LLINs should be effectively communicated to the community, ensuring that it is both understood and acknowledged by the target population. Further research is needed to identify the factors that support the sustained use of LLINs in a way that is acceptable and understood by the community.

## Supplementary Information

Supplementary Material 1.

## Data Availability

No datasets were generated or analysed during the current study.

## Abbreviations

AIC: Akaike Information Criterion
β: Beta coefficient
GLM: Generalized linear model
IRS: Indoor residual spraying
IVM: Integrated vector control management
LLINs: Long-lasting insecticide-treated nets
MCA: Multiple correspondence analysis
OR: Odd ratios
PCR: Polymerase chain reaction
SE: Standard Error
SES: Socioeconomic status
VIF: Variance inflation factors
