# Supplementary material for: Long-lasting insecticidal net ownership and malaria infection by socio-economic status: a cross-sectional household study in an area along Lake Victoria, Kenya
Source: Malar J. 2025 Oct 22;24:355. doi: 10.1186/s12936-025-05528-x (PMC12541966; doi:10.1186/s12936-025-05528-x)
Supplement: Supplementary file 1 — Supplementary Material 1. [file 12936_2025_5528_MOESM1_ESM.docx]

**Supplementary table 1: Characteristics of individuals**

**Supplementary table 2:** **Information used to categorize the SES**

**Supplementary table 3: Details of malaria types in households with malaria-positive children**

**Supplementary table 4: Parameter estimates for the GLM explaining malaria infection status in the absence of interaction terms (N=705)**

| Variables^1^ | Adjusted OR | 95% CI |
| --- | --- | --- |
| LLIN ownership  (ref. insufficient) | 0.59 | 0.30-1.24 |
| SES_middle (ref. high) | 1.10 | 0.62-1.94 |
| SES_low (ref. high) | 0.89 | 0.43-1.75 |
| IRS_conducted (ref. No) | 1.22 | 0.73-2.05 |
| Eave (ref. opened) | 0.60 | 0.22-1.37 |
| Wall (ref. mud) | 1.05 | 0.56-1.99 |
| Floor (ref. mud) | 0.59 | 0.32-1.08 |

^1^All VIF values are lower than 1.61.

**Supplementary table 5: Malaria infection status of the household for each variable**
